# Lymphocyte Membrane‐ and 12p1‐Dual‐Functionalized Nanoparticles for Free HIV‐1 Trapping and Precise siRNA Delivery into HIV‐1‐Infected Cells

**DOI:** 10.1002/advs.202300282

**Published:** 2023-02-08

**Authors:** Jinbang Zhang, Jingwan Han, Hui Li, Zhengyang Li, Pengfei Zou, Jiaxin Li, Te Zhao, Junwei Che, Yang Yang, Meiyan Yang, Yuli Wang, Wei Gong, Zhiping Li, Lin Li, Chunsheng Gao, Haihua Xiao

**Affiliations:** ^1^ State key Laboratory of Toxicology and Medical Countermeasure Department of Pharmaceutics Beijing Institute of Pharmacology and Toxicology Beijing 100039 China; ^2^ Pharmaceutical College Henan University Kaifeng 475001 China; ^3^ State Key Laboratory of Pathogen and Biosecurity Beijing Institute of Microbiology and Epidemiology Beijing 100071 China; ^4^ School of Public Health and Health Management Gannan Medical University Ganzhou 341000 China; ^5^ Institute of Chemistry Chinese Academy of Sciences Beijing 100190 China

**Keywords:** 12p1, human immunodeficiency virus, lipid nanoparticles, lymphocyte membrane, siRNA

## Abstract

Despite the success of small interfering RNA (siRNA) in clinical settings and its potential value in human immunodeficiency virus (HIV) therapy, the rapid clearance and absence of precise delivery to target cells still hinder the therapeutic effect of siRNA. Herein, a new system, which can escape immune recognition, has HIV‐1 neutralizing capacity, and the ability to deliver siRNA specifically into HIV‐1‐infected cells, is constructed by functionalizing siRNA delivery lipid nanoparticles with the lymphocyte membrane and 12p1. The constructed system is shown to escape uptake by the mononuclear phagocyte system. The constructed system exhibits strong binding ability with gp120, thus displaying distinguished neutralizing breadth and potency. The constructed system neutralizes all tested HIV‐1 pseudotyped viruses with a geometric mean 80% inhibitory concentration (IC80) of 29.75 µg mL^−1^ and inhibits X4‐tropic HIV‐1 with an IC80 of 64.20 µg mL^−1^, and R5‐tropic HIV‐1 with an IC80 of 16.39 µg mL^−1^. The new system also specifically delivers siRNA into the cytoplasm of HIV‐1‐infected cells and exhibits evident gene silencing of *tat* and *rev*. Therefore, this new system can neutralize HIV‐1 and deliver siRNA selectively into HIV‐1‐infected cells and may be a promising therapeutic candidate for the precise therapy of HIV.

## Graphical abstract

Lymphocyte membrane‐ and 12p1‐dual‐functionalized nanoparticles are constructed for free HIV‐1 trapping and precise siRNA delivery into HIV‐1‐infected cells. This new system exhibits strong binding ability with gp120, distinguished neutralizing potency against HIV‐1, and evident gene silencing of tat and rev in HIV‐1‐infected cells. Therefore, the constructed system can neutralize HIV‐1 and deliver siRNA selectively into the cytoplasm of HIV‐1‐infected cells.

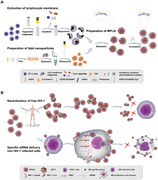

## 1 Introduction

Despite decades of research, acquired immune deficiency syndrome remains a major public health problem worldwide, with more than 38 million people affected and an increasing number of new infections.^[^ ^1^ ^]^ Highly active antiretroviral therapy (HAART) is the current mainstay of human immunodeficiency viruses (HIV) therapy, and its success in fully suppressing viral replication and prolonging the life expectancy of HIV‐1‐infected individuals has been verified.^[^ ^2^ ^]^ However, low oral bioavailability, poor drug‐regimen compliance, increased side effects as a consequence of the high and frequent dosages, and high costs severely affect the therapeutic results of HAART against HIV.^[^ ^2^, ^3^ ^]^ Therefore, alternative therapeutic options and strategies against HIV are needed.^[^ ^2^ ^]^

Nucleic acid‐based therapeutics, such as antisense oligonucleotides, aptamers, and RNA interference (RNAi), have emerged as alternatives to HAART.^[^ ^2^ ^]^ RNAi, one of the most potent nucleic acid‐based approaches, has become popular due to its potential to mediate sequence‐specific post‐transcriptional gene silencing.^[^ ^2^ ^]^ HIV‐1 was the first infectious agent to be targeted by RNAi due to a better understanding of the gene expression patterns in the HIV‐1 life cycle.^[^ ^4^ ^]^ RNAi holds the potential to overcome the high rate of HIV mutation by targeting highly conserved regions on viral genes, which are important for viral replication.^[^ ^2^, ^5^ ^]^ Small interfering RNA (siRNA), short double‐stranded RNA of 21–23 nucleotides, is the most commonly used RNAi tool. siRNAs can target particular genes to induce short‐term highly specific silencing to block the production of the respective proteins.^[^ ^6^ ^]^ Several siRNA therapeutics have been approved for the market. siRNAs silencing major HIV‐1 regulatory genes, such as *tat*, *rev*, *nef*, and *vif*, are also being developed, and those genes simultaneously encoding two proteins are believed to be more potent.^[^ ^4^, ^7^ ^]^ However, siRNAs only play a therapeutic role in the cytoplasm, and their rapid degradation by RNase and the low cell permeability due to their strong hydrophilicity and inherently strong negative charge hamper their entry into cells.^[^ ^2^, ^4^, ^6^ ^]^ Therefore, considerable efforts have been made to develop effective siRNA delivery carriers,^[^ ^2^, ^4^, ^6^ ^]^ including nanocarriers, liposomes, polymeric nanoparticles, dendrimers, quantum rods, carbon nanotubes, and inorganic nanoparticles.^[^ ^2^, ^4^, ^6^ ^]^ Among these, lipid‐based vectors are considered one of the most promising carriers and several lipid‐based products have been applied in clinical settings.

Despite major achievements, some problems, such as nonspecific distribution of the delivery system, clearance by the mononuclear phagocyte system (MPS), and low endosomal escape efficiency, are known to seriously affect the efficiency of siRNA delivery into the cytoplasm of target cells.^[^ ^2^, ^6^ ^]^ To increase the targeting ability to free HIV virions or HIV‐infected cells, new delivery systems have been designed to encompass conjugation with PEGylated targeting moieties, such as antibodies, aptamers, and other ligands.^[^ ^2^, ^3^, ^7^, ^8^ ^]^ Several studies have consistently shown that the conjugation of nanoparticles with a targeting ligand increases the association of the nanoparticles with target cells, thereby increasing the specificity of drug delivery and concurrently reducing off‐target effects and toxicity.^[^ ^7^, ^9^ ^]^ Although PEGylation is one of the major approaches for imparting stealthiness to these targeted drug delivery systems, the unexpected clearance of PEGylated materials in vivo after repeated administration remains a limitation to be overcome.^[^ ^10^ ^]^

Biomimetic technology, especially coating with the cell membrane—an emergent alternative to PEGylation—meets these needs and is actively used in targeted nanomedicines.^[^ ^1^, ^6^, ^8^, ^10^ ^]^ Coating with cell membrane facilitates the transfer of membrane proteins, including receptors from source cells, onto the nanoparticle surface, thereby producing many distinct advantages, such as reduced MPS clearance, prolonged blood circulation, and improved accumulation at specific pathological sites.^[^ ^1^, ^6^, ^8^, ^11^ ^]^ It has even been inferred that nanocarriers made with the host cell membrane will be able to neutralize the infection, provide a broad‐acting countermeasure resistant to mutations, and protect against viruses as long as the target of the virus remains the identified host cells.^[^ ^9^ ^]^

Several T cell‐mimicking nanotraps and nanoparticles have been developed, and their neutralization against free HIV and targeting of HIV‐infected cells have been authenticated.^[^ ^1^, ^3^, ^12^ ^]^ However, the infection‐inhibiting efficacy of these cellular membrane‐based nanoscale vesicles is limited^[^ ^13^ ^]^ due to the high number of host cells in vivo, which makes it difficult for the definite cellular membrane‐based vesicles to competitively bind with free viruses or virus‐infected cells on infinite host cells. Therefore, the targeting design of vesicles coated with cell membranes should be improved to enhance their competitive binding ability with free viruses or virus‐infected cells.

Decoration with a certain density of ligands on cellular membrane‐based nanoparticles might enhance their binding probability with free viruses or virus‐infected cells, facilitate the precise delivery of siRNA into virus‐infected cells, and finally improve the infection‐inhibiting efficacy. The linear peptide,12p1 (RINNIPWSEAMM), has been shown to preferentially bind with gp120 prior to clusters of differentiation 4 (CD4) or chemokine receptor 5 (CCR5), and block the binding of gp120 to the native coreceptor CD4 or CCR5.^[^ ^14^ ^]^ Currently, 12p1 has been used as a CD4‐gp120 inhibitor and the peptide probe of the gp120 structure.^[^ ^15^ ^]^ Therefore, 12p1 is considered a candidate targeting the ligand of gp120. The combination of 12p1 with the T cell membrane may predominantly enhance the nanoparticles’ competitiveness of binding with gp120 compared to native T cells and may show satisfactory infection‐inhibiting efficacy.

**Figure 1:**
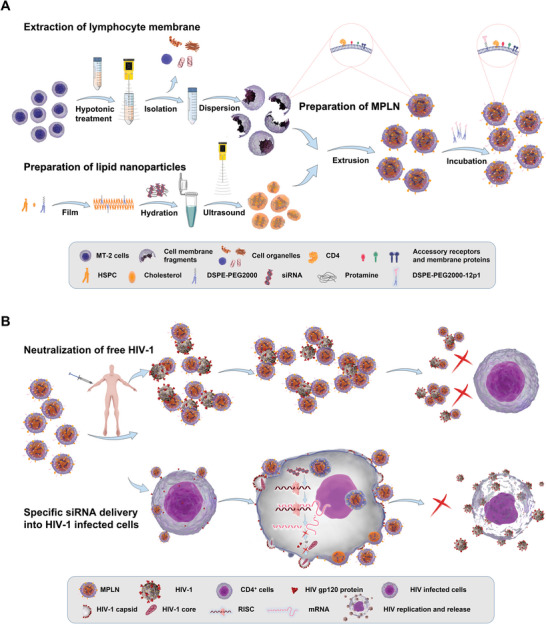
Preparation and proposed illustration of the lymphocyte membrane‐ and 12p1‐dual functionalized siRNA delivery lipid nanoparticle system (MPLN). A) Schematic of MPLN preparation. B) Proposed schematic illustration of MPLN.

Accordingly, a lymphocyte membrane‐ and 12p1‐dual functionalized siRNA delivery lipid nanoparticle system (MPLN) was constructed as shown in **Figure** **1**A, and the schematic illustration of MPLN is proposed in Figure 1B. After injection, MPLN first escapes from MPS uptake and circulates throughout the whole body due to the existence of CD47 from the cell membrane and the inserted polyethylene glycol (PEG). Then, MPLN targets gp120 expressed by free HIV virions or HIV‐infected cells and binds to them under the guidance of the T cell membrane and 12p1; as a result, MPLN can directly neutralize free HIV and inhibit gp120‐induced killing of bystander cells. In parallel, siRNA encapsulated in MPLN can be specifically delivered into HIV‐infected cells, where it serves to suppress HIV replication. Therefore, significantly improved HIV inhibition is expected for this dual‐functionalized siRNA delivery system.

To validate the HIV‐inhibition efficacy of MPLN, the human lymphoma (MT2) cell membrane and 12p1 functionalized lipid nanoparticles containing siRNA targeting HIV‐1 *tat* and *rev* transcripts were constructed, and their characterization, ability to bind to gp120, HIV‐1 neutralization, ability to deliver siRNA into the cytoplasm of HIV‐1‐infected cells, inhibition of gp120‐induced bystander cell killing, distribution, and safety in vivo were investigated.

## 2 Results

### 2.1 Characterization of the Constructed Nanoparticles

**Figure 2:**
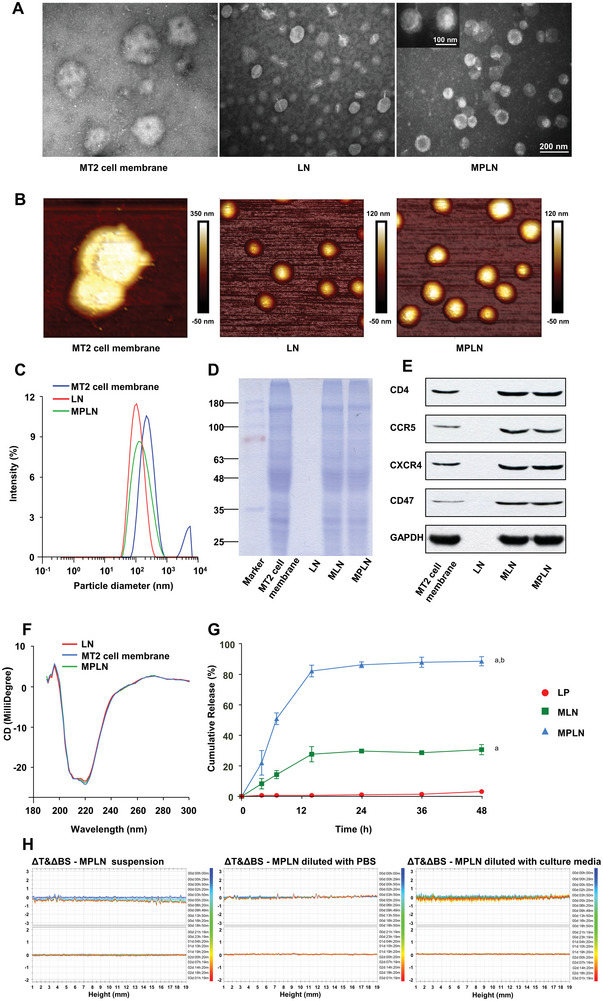
Characteristics of various nanoparticles. A) transmission electron microscopy (TEM) images. The size marker for MPLN (200 nm) also applied to the MT2 cell membrane and LN. B) Plane surface images from atomic force microscopy (AFM). C) Intensity size distributions of the MT2 cell membrane, LN, MLN, and MPLN via dynamic light scattering at 25 °C. The determining for all samples was conducted with 11 cycles. D) Protein profiles determined using sodium dodecyl sulfate‐polyacrylamide gel electrophoresis assay. E) Identification of specific proteins, including CD4, CCR5, CXCR4, and CD47, analyzed using Westen Blot (WB) assay. F) The average far‐ultraviolet circular dichroism spectra recorded with a wavelength range of 190–300 nm and a bandwidth of 5 nm at 37 °C. *n* = 3. G) In vitro release profiles of cyanine 5 labeled siRNA (Cy5‐siRNA) from LN, MLN, and MPLN. The experiments were inspected in phosphate‐buffered saline (PBS, pH 7.4) using a dialysis technique at 37 °C. The analysis was performed using a spectrofluorometer with 649 nm as the excitation wavelength and 680 nm as the emission wavelength. Data were presented as mean ± SD, *n* = 3, *p*‐values are calculated using the two‐sample *t*‐test. a: *p* < 0.05 versus LN; b: *p* < 0.05 versus MLN. H) Colloidal stability analysis of MPLN, MPLN diluted 100‐fold with PBS (pH 7.4), and MPLN diluted 100‐fold with cell culture media containing fetal bovine serum (FBS, 10%, W/V) at 37 °C.

MPLN and lipid nanoparticles containing siRNA (LN) appeared consistently round, while the extracted MT2 cell membrane was irregular under both transmission electron microscopy (TEM) and atomic force microscopy (AFM), as shown in **Figure** **2**A,B. The particle sizes of the MT2 cell membrane, LN, and MPLN (Figure 2C) were 236.60 ± 50.25, 98.67 ± 16.01, and 129.70 ± 18.38 nm, respectively, with a dispersity index of 0.344, 0.178, and 0.233, respectively. The zeta potentials of the MT2 cell membrane, LN, and MPLN were −22.5 ± 0.6, −10.7 ± 0.2, and −16.1 ± 0.6 mV, respectively. The encapsulation efficiencies of siRNA were 97.36% ± 1.14% and 92.13% ± 0.96% for LN and MPLN, respectively.

To verify the transfer of proteins from the cell membrane to MPLN, the protein profile, specific proteins, and secondary structure of proteins from the extracted MT2 cell membrane and different nanoparticles were detected, and the results are shown in Figure 2D–F. There were no significant differences in the protein profile of the extracted MT2 cell membrane and MPLN, and no protein signal was detected in LN. Specific proteins, including CD4, CCR5, chemokine (C‐X‐C motif) receptor 4 (CXCR4), and CD47, were displayed in the purified MT2 cell membrane and MPLN, but not in LN. There was a positive band at 196 nm and a negative band at 220 nm in all far‐ UV circular dichroism spectra (CD), and no significant shifts in the peaks or obvious changes in peak intensity.

The release of siRNA from LN, the MT2 cell membrane‐modified lipid nanoparticles (MLN), and MPLN was tested as shown in Figure 2G. It was obvious that siRNA was released slowly from MPLN or MLN, and the cumulative release of siRNA from MPLN could reach about 90% at 48 h while there was only about 30% of siRNA released from MLN at 48 h. In addition, there seemed to be no siRNA released from LN.

The colloidal stability of MPLN and its dilutions with phosphate‐buffered saline (PBS) or cell culture media containing 10% fetal bovine serum (FBS) was monitored using Turbiscan Lab Expert, and the results are shown in Figure 2F. Additionally, the variations in both transmission and backscattering were less than 1% for all samples over a 72‐h period.

### 2.2 In Vitro Cellular Uptake

**Figure 3:**
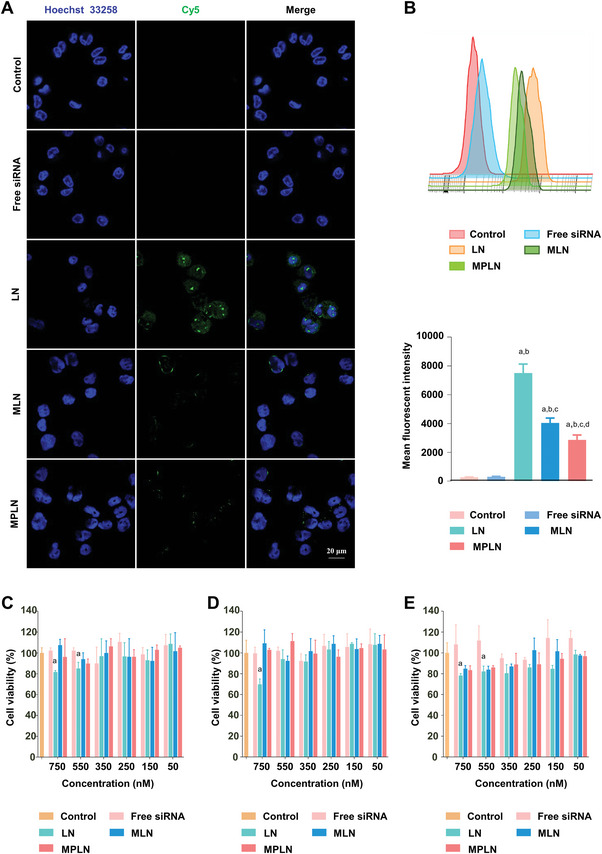
Cellular evaluation of MPLN. A) Cellular uptake evaluation of different nanoparticles at a Cy5‐siRNA concentration of 250 nM by macrophages derived from THP‐1 cells under confocal laser scanning microscopy (CLSM). Hoechst 33258 for nuclear staining (blue) and Cy5 fluorescence (green) are shown. The scale bar is 20 µm for all images. B) Cellular uptake evaluation of different nanoparticles at a Cy5‐siRNA concentration of 250 nM by macrophages derived from THP‐1 cells using flow cytometry (FCM). Data were presented as mean ± SD, *n* = 3, *p*‐values are calculated using the two‐sample *t*‐test. a: *p* < 0.05 versus Control; b: *p* < 0.05 versus free siRNA; c: *p* < 0.05 versus LN; d: *p* < 0.05 versus MLN. C) Cell viability assay of MT2 cells treated with different nanoparticles at siRNA concentrations of 50, 150, 250, 350, 550, and 750 nM for 24 h. Data were presented as mean ± SD, *n* = 3, *p*‐values are calculated using the two‐sample *t*‐test. D) Cell viability assay of TZM‐bl cells treated with different nanoparticles at siRNA concentrations of 50, 150, 250, 350, 550, and 750 nM for 24 h. Data were presented as mean ± SD, *n* = 3, *p*‐values are calculated using the two‐sample *t*‐test. E) Cell viability assay of PBMC treated with different nanoparticles at siRNA concentrations of 50, 150, 250, 350, 550, and 750 nM for 24 h. Data were presented as mean ± SD, *n* = 3, *p*‐values are calculated using the two‐sample *t*‐test.

The uptake of nanoparticles by human macrophages differentiated from human myeloid leukemia mononuclear (THP‐1) cells was analyzed using both confocal laser scanning microscopy (CLSM) and flow cytometry (FCM), and the results are exhibited in **Figure** **3**A,B. There was almost no fluorescence observed in cells treated with PBS control or free siRNA. In contrast, intensive fluorescence was found in cells treated with LN, and there was slight fluorescence displayed in cells treated with MLN or MPLN. The results from FCM were coincident with those from CLSM, in that macrophages treated with LN displayed the highest fluorescence intensity, followed by MLN, MPLN, and free siRNA or control. Significant differences in the fluorescence intensity were found between LN and MLN, LN and MPLN, free siRNA and MLN, free siRNA and MPLN, and MLN and MPLN.

### 2.3 Cytotoxicity

The cytotoxicity of the nanoparticles on TZM‐bl cells, MT2 cells, and peripheral blood mononuclear cells (PBMC) was assessed by analyzing cell viability with the Cell Counting Kit‐8 (CCK‐8) assay. As shown in Figure 3C−E, there was no significant change found in cell viability for all three cell lines treated with MLN or MPLN compared with control while the viability of all three cell lines treated with LN significantly decreased compared with control when the concentration of LN used was more than 550 nM (siRNA concentration) or 1.84 mg mL^−1^ (nanoparticle mass concentration).

### 2.4 Binding of MPLN with gp120

**Figure 4:**
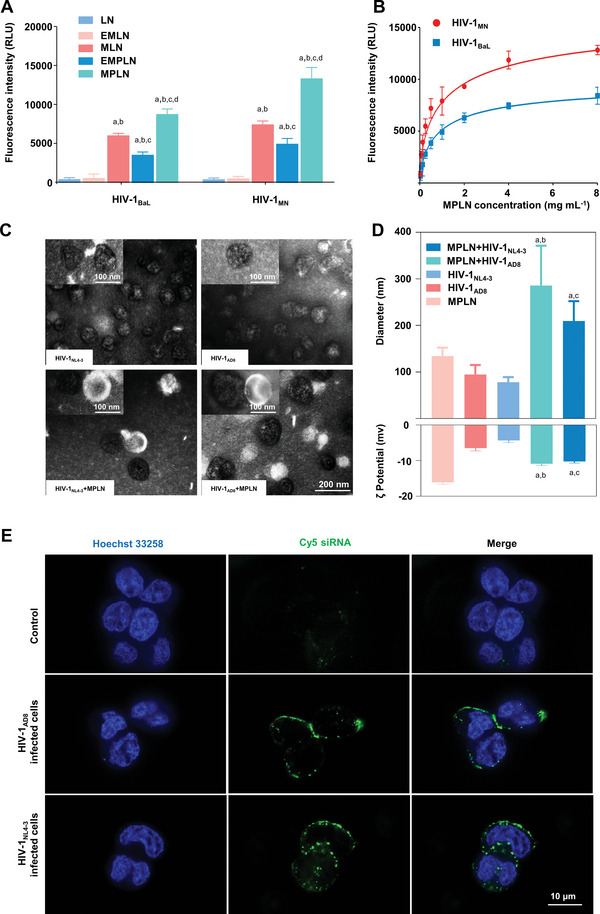
Binding capacity and specificity of MPLN against the HIV‐1 envelope glycoprotein gp120. A) Binding ability of Cy5 labeled LN, EMLN, MLN, EMPLN, and MPLN with R5‐tropic HIV‐1_BaL_ gp120 recombinant protein and X4‐tropic HIV‐1_MN_ gp120 recombinant protein, respectively. The evaluating index was the fluorescence intensity of samples at 649 nm with an emission wavelength of 680 nm. Data were presented as mean ± standard derivation, *n* = 3, *p*‐values are calculated using the two‐sample *t*‐test. a: *p* < 0.05 versus LN; b: *p* < 0.05 versus EMLN; c: *p* < 0.05 versus MLN; d: *p* < 0.05 versus EMPLN. B) Binding profiles of MPLN at the concentrations of 0.125, 0.25, 0.5, 1, 2, 4, and 8 mg mL^−1^ with R5‐tropic HIV‐1_BaL_ gp120 recombinant protein and X4‐tropic HIV‐1_MN_ gp120 recombinant protein, respectively. Data were presented as mean ± SD, *n* = 3. C) TEM images of HIV‐1 virion, MPLN, and a mixture of HIV‐1 virion and MPLN. The scale bar is 200 nm for the four large images. D) Particle size and surface zeta potential of HIV‐1 virion, MPLN, and their mixtures. Data were presented as mean ± SD, *n* = 3, *p*‐values are calculated using the two‐sample *t*‐test. a: *p* < 0.05 versus MPLN; b: *p* < 0.05 versus HIV‐1_AD8_; c: *p* < 0.05 versus HIV‐1_NL4‐3_. E) Binding of MPLN with HIV‐1_AD8_‐infected cells and HIV‐1_NL4‐3_‐infected cells under CLSM. The scale bar is 10 µm for all images.

The binding capability of MPLN with different HIV‐1 gp120 recombinant proteins, including, X4‐tropic HIV‐1_MN_ gp120 and R5‐tropic HIV‐1_BaL_ gp120, was first investigated. The binding capability of various nanoparticles is displayed in **Figure** **4**A, and the binding curves of MPLN with gp120 recombinant proteins are shown in Figure 4B. Very low fluorescence intensity was found in the samples treated with LN or the erythrocyte membrane‐modified lipid nanoparticles (EMLN) regardless of gp120 type. The fluorescence intensity was obviously improved for gp120 proteins incubated with MLN, the erythrocyte membrane, and 12p1 dual modified lipid nanoparticles (EMPLN) or MPLN, and the strongest fluorescence intensity was displayed in the group treated with MPLN. The fluorescence intensity of the captured MPLN increased gradually with increasing MPLN concentration, and the plateau was reached when the concentration of MPLN was approximately 4 mg mL^−1^ for both HIV‐1_MN_ gp120 and HIV‐1_BaL_ gp120. A Langmuir binary interaction model was used to fit the data curves, and the calculated dissociation constant was 0.83 ± 0.39 mg mL^−1^ for immobilized HIV‐1_MN_ gp120 and 1.05 ± 0.22 mg mL^−1^ for immobilized HIV‐1_BaL_ gp120.

The binding ability of MPLN with HIV‐1 envelope glycoprotein was further evaluated by monitoring the characteristic changes in morphology, particle size, and zeta potential. X4‐tropic HIV‐1_NL4‐3_ and R5‐tropic HIV‐1_AD8_ were used as model viruses. As shown in Figure 4C, TEM revealed the aggregation of MPLN at the surface of virus particles. The particle size of the mixtures of HIV‐1_NL4‐3_ and MPLN increased to 209.43 ± 42.87 from 77.97 ± 11.20 nm for HIV‐1_NL4‐3_ and 134.40 ± 18.11 nm for MPLN (Figure 4D), and the zeta potential of the mixtures of HIV‐1_NL4‐3_ and MPLN changed to −10.24 ± 0.44 from −4.35 ± 0.60 mV for HIV‐1_NL4‐3_ and −16.10 ± 0.61 mV for MPLN (Figure 4D). Similar changes were found for the mixtures of HIV‐1_AD8_ and MPLN; that is, the particle size of the mixtures of HIV‐1_AD8_ and MPLN increased to 285.87 ± 85.42 from 94.67 ± 20.49 nm for HIV‐1_AD8_ and 134.40 ± 18.11 nm for MPLN, and the zeta potential of the mixtures of HIV‐1_AD8_ and MPLN changed to −10.90 ± 0.46 from −6.50 ± 0.78 mV for HIV‐1_NL4‐3_ and −16.10 ± 0.61 mV for MPLN.

To evaluate the binding ability and the selectivity of MPLN to HIV‐1‐infected cells, HIV‐1_NL4‐3_‐ and HIV‐1_AD8_‐infected TZM‐bl cells were used as model cells, and the binding efficacy was observed under CLSM, as shown in Figure 4E. As expected, negligible fluorescence was found in uninfected cells (control), and significantly stronger fluorescence was observed in both types of infected cells treated with MPLN.

### 2.5 HIV Neutralization Assay

**Figure 5:**
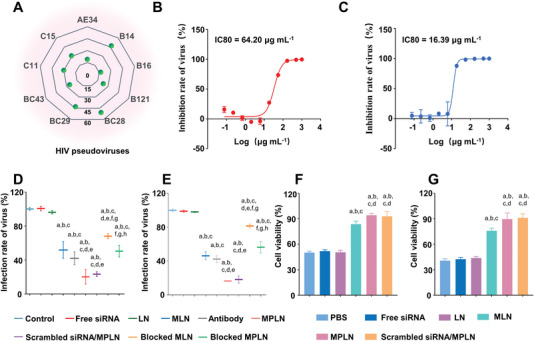
Efficacy evaluation of MPLN. A) IC80 (µg mL^−1^) of MPLN against different HIV env‐pseudoviruses. Nine geographically and genetically diverse env‐pseudoviruses (AE34, B14, B16, B121, BC28, BC29, BC43, C11, and C15) representing the main subtypes and recombinant forms circulating in China were used. The measurements are conducted in triplicate. B) Inhibition curve of X4‐tropic HIV‐1_NL4‐3_ infection by MPLN. Data were presented as mean ± SD, *n* = 3. Dose–response curves were fitted using nonlinear regression, and IC80 values were calculated using GraphPad Prism 8. C) Inhibition curve of R5‐tropic HIV‐1_AD8_ infection by MPLN. Data were presented as mean ± SD, *n* = 3. Dose–response curves were fitted using nonlinear regression, and IC80 values were calculated using GraphPad Prism 8. D) Inhibition of LN, MLN, MPLN, scrambled siRNA/MPLN, the antibody mixtures of CD4, CXCR4, and CCR5, the antibody‐blocked MLN, and the antibody‐blocked MPLN on X4‐tropic HIV‐1_NL4‐3_ infection. Data were presented as mean ± SD, *n* = 3, *p*‐values are calculated using the two‐sample *t*‐test. a: *p* < 0.05 versus Control; b: *p* < 0.05 versus free siRNA; c: *p* < 0.05 versus LN; d: *p* < 0.05 versus MLN; e: *p* < 0.05 versus antibody mixtures; f: *p* < 0.05 versus MPLN; g: *p* < 0.05 versus scrambled siRNA/MPLN; h: *p* < 0.05 versus antibody blocked MLN. E) Inhibition of LN, MLN, MPLN, scrambled siRNA/MPLN, the antibody mixtures of CD4, CXCR4, and CCR5, the antibody‐blocked MLN, and the antibody‐blocked MPLN on R5‐tropic HIV‐1_AD8_ infection. Data were presented as mean ± SD, *n* = 3, *p*‐values are calculated using the two‐sample *t*‐test. a: *p* < 0.05 versus Control; b: *p* < 0.05 versus free siRNA; c: *p* < 0.05 versus LN; d: *p* < 0.05 versus MLN; e: *p* < 0.05 versus antibody mixtures; f: *p* < 0.05 versus MPLN; g: *p* < 0.05 versus scrambled siRNA/MPLN; h: *p* < 0.05 versus antibody blocked MLN. F) Inhibition of LN, MLN, MPLN, and scrambled siRNA/MPLN on X4‐tropic HIV‐1_MN_ gp120‐induced human naive CD4^+^ T cells killing. Data were presented as mean ± standard derivation, *n* = 3, *P*‐values are calculated using the two‐sample *t*‐test. a: *p* < 0.05 versus PBS; b: *p* < 0.05 versus free siRNA; c: *p* < 0.05 versus LN; d: *p* < 0.05 versus MLN. G) Inhibition of LN, MLN, MPLN, and scrambled siRNA/MPLN on R5‐tropic HIV‐1_BaL_ gp120‐induced human naive CD4^+^ T cells killing. Data were presented as mean ± SD, *n* = 3, *p*‐values are calculated using the two‐sample *t*‐test. a: *p* < 0.05 versus PBS; b: *p* < 0.05 versus free siRNA; c: *p* < 0.05 versus LN; d: *p* < 0.05 versus MLN.

To evaluate the neutralizing breadth and potency of MPLN against HIV‐1, nine strains of geographically and genetically diverse envelope glycoprotein (env)‐pseudotype viruses were used. The neutralizing breadth of MPLN was 100% against the combined nine‐virus panel, and the geometric mean 50% inhibitory concentration (IC50) and the geometric mean 80% inhibitory concentration (IC80) of MPLN against all nine viral strains were 18.33 and 29.75 µg mL^−1^, respectively, as shown in **Figure** **5**A.

The inhibitory effect of MPLN against X4‐tropic HIV‐1_NL4‐3_ and R5‐tropic HIV‐1_AD8_ was also evaluated on TZM‐bl cells with HIV p24 antigen production as an index, and the results are shown in Figure 5B,C. MPLN evidently decreased the p24 levels of both HIV‐1 strains. The values of IC50 and IC80 were 33.91 and 64.20 µg mL^−1^, respectively, for MPLN neutralizing X4‐tropic HIV‐1_NL4‐3_ strain, and 12.23 and 16.39 µg mL^−1^, respectively for MPLN inhibiting R5‐tropic HIV‐1_AD8_ strain.

To further confirm the primary action of the MT2 cell membrane coating and 12p1 decoration, the neutralizing efficacy of LN, MLN, scrambled siRNA/MPLN, and the mixtures of various nanoparticles with antibodies was evaluated. As displayed in Figure 5D, there were almost no HIV‐1_NL4‐3_‐neutralizing effects for free siRNA and LN, while significant HIV‐1_NL4‐3_‐neutralizing efficacy was shown for MLN, MPLN, scrambled siRNA/MPLN, and antibodies. The most pronounced HIV‐1_NL4‐3_ neutralization was observed in MPLN and scrambled siRNA/MPLN, followed by antibodies, MLN, blocked MPLN, and blocked MLN. Significant differences in neutralizing efficacy were found between MLN and LN, MPLN and LN, scrambled siRNA/MPLN and LN, and MPLN and MLN. However, there were no significant differences in neutralizing efficacy between MPLN and scrambled siRNA/MPLN. Similar results were exhibited in terms of HIV‐1_AD8_‐neutralizing efficacy, as shown in Figure 5E.

### 2.6 Inhibition of HIV‐1 gp120‐Induced Bystander T‐Cell Killing

The potential of MPLN for neutralizing the cytotoxicity of gp120 was also evaluated. Approximately 40% of the isolated CD4^+^ T cells died following treatment with X4‐tropic HIV‐1_MN_ gp120 recombinant proteins as displayed in Figure 5F. The introduction of free siRNA or LN in combination with HIV‐1_MN_ gp120 recombinant proteins had no significant influence on the viability of CD4^+^ T cells. However, the viability of CD4^+^ T cells significantly improved following treatment with the mixtures of HIV‐1_MN_ gp120 recombinant proteins and MLN or MPLN, and the highest viability of CD4^+^ T cells was found in the groups treated with the mixtures of HIV‐1_MN_ gp120 recombinant proteins and MPLN regardless of siRNA. The inhibition efficacy of MPLN against R5‐tropic HIV‐1_BaL_ gp120 recombinant proteins was also tested, with the results shown in Figure 5G. Similar to the results against HIV‐1_BaL_ gp120 recombinant proteins, MPLN and scrambled siRNA/MPLN displayed the strongest inhibition of gp120‐induced CD4^+^ T‐cell death, followed by MLN, and neither free siRNA nor LN showed any evident inhibition of gp120‐induced CD4^+^ T‐cell death.

### 2.7 Endosomal Escape

**Figure 6:**
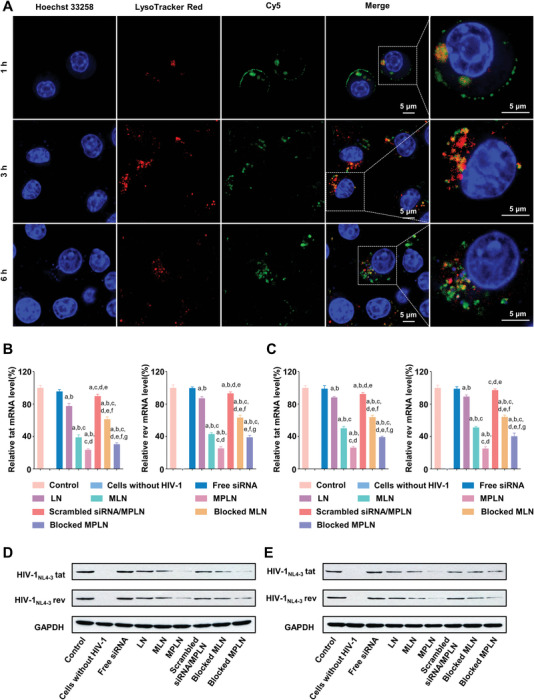
siRNA delivery of MPLN into HIV‐1‐infected cells and the resultant gene‐silencing efficacy. A) Endosomal escape of siRNA in MPLN under CLSM. RAW264.7 cells were used as the model cell. siRNA was labeled with Cy5 (green), endolysosomes in the cells were stained with LysoTracker Red (red), and nuclei were stained with Hoechst 33258 (blue). The scale bar is 5 µm for all images. B) Levels of *tat* and *rev* mRNA in HIV‐1_NL4‐3_‐infected cells treated with LN, MLN, MPLN, scrambled siRNA/MPLN, antibody‐blocked MLN, and antibody‐blocked MPLN. The level of mRNA was analyzed on a real‐time fluorescence quantitative PCR detection system. SYBR Green was used. Data were presented as mean ± SD, *n* = 3, *p*‐values are calculated using the two‐sample *t*‐test. a: *p* < 0.05 versus Control; b: *p* < 0.05 versus free siRNA; c: *p* < 0.05 versus LN; d: *p* < 0.05 versus MLN; e: *p* < 0.05 versus MPLN; f: *p* < 0.05 versus scrambled siRNA/MPLN; g: *p* < 0.05 versus antibody‐blocked MLN. C) Levels of *tat* and *rev* mRNA in HIV‐1_AD8_‐infected cells treated with LN, MLN, MPLN, scrambled siRNA/MPLN, antibody‐blocked MLN, and antibody‐blocked MPLN. The level of mRNA was analyzed on a real‐time fluorescence quantitative PCR detection system. SYBR Green was used. Data were presented as mean ± SD, *n* = 3, *p*‐values are calculated using the two‐sample *t*‐test. a: *p* < 0.05 versus Control; b: *p* < 0.05 versus free siRNA; c: *p* < 0.05 versus LN; d: *p* < 0.05 versus MLN; e: *p* < 0.05 versus MPLN; f: *p* < 0.05 versus scrambled siRNA/MPLN. g: *p* < 0.05 versus antibody‐blocked MLN. D) Levels of *tat* and *rev* protein expression in HIV‐1_NL4‐3_‐infected cells treated with LN, MLN, MPLN, scrambled siRNA/MPLN, antibody‐blocked MLN, and antibody‐blocked MPLN. Protein expression was detected with the WB method. E) Levels of *tat* and *rev* protein expression in HIV‐1_AD8_‐infected cells treated with LN, MLN, MPLN, scrambled siRNA/MPLN, antibody‐blocked MLN, and antibody‐blocked MPLN. Protein expression was detected with the WB method.

The endosomal escape of MPLN was visualized using CLSM. The distribution of siRNA and endolysosomes were identified as green and red fluorescence, respectively, given that siRNA was labeled with cyanine 5 (Cy5) and endolysosomes were stained with LysoTracker Red before imaging. The confluence of green and red fluorescence was shown as yellow; that is, the presence of yellow fluorescence indicated that siRNA was entrapped within endolysosomes. As shown in **Figure** **6**A, some green fluorescence at the cell surface and some yellow fluorescence were visible 1 h after the addition of nanoparticles. After 3 h, the green fluorescence at the cell surface almost disappeared, while the yellow fluorescence and the red fluorescence accounted for most of the fluorescent signal. The green fluorescence seemed to gradually dominate at 6 h in cells treated with MPLN.

### 2.8 In Vitro Gene Silencing

To verify the gene‐silencing effect of siRNA, the *tat* and *rev* messenger RNA (mRNA) levels were analyzed with a real‐time fluorescence quantitative polymerase chain reaction (PCR) system, and *tat* and *rev* protein expression levels were detected using western blot (WB) analysis. As shown in Figure 6B, there was no evident *tat* or *rev* mRNA suppression in HIV‐1_NL4‐3_‐infected cells treated with free siRNA, while slight *tat* and *rev* suppression was shown in HIV‐1_NL4‐3_‐infected cells treated with LN or scrambled siRNA/MPLN. Obvious *tat* and *rev* mRNA suppression was observed in other formulations. The efficiency of *tat* and *rev* mRNA suppression in HIV‐1_NL4‐3_‐infected cells decreased in the following order: MPLN, blocked MPLN, MLN, blocked MLN, and LN. Consistent results were also exhibited in HIV‐1_AD8_‐infected cells, as displayed in Figure 6C.

Similar to the suppression of *tat* and *rev* mRNA, downregulation of *tat* and *rev* proteins was also found in both HIV‐1_NL4‐3_‐infected cells and HIV‐1_AD8_‐infected cells treated with nanoparticles of different formulations, except those treated with free siRNA (Figure 6D,E). The lowest *tat* and *rev* protein expression levels were found in cells treated with MPLN, regardless of HIV strains.

### 2.9 In Vivo Safety Evaluation

**Figure 7:**
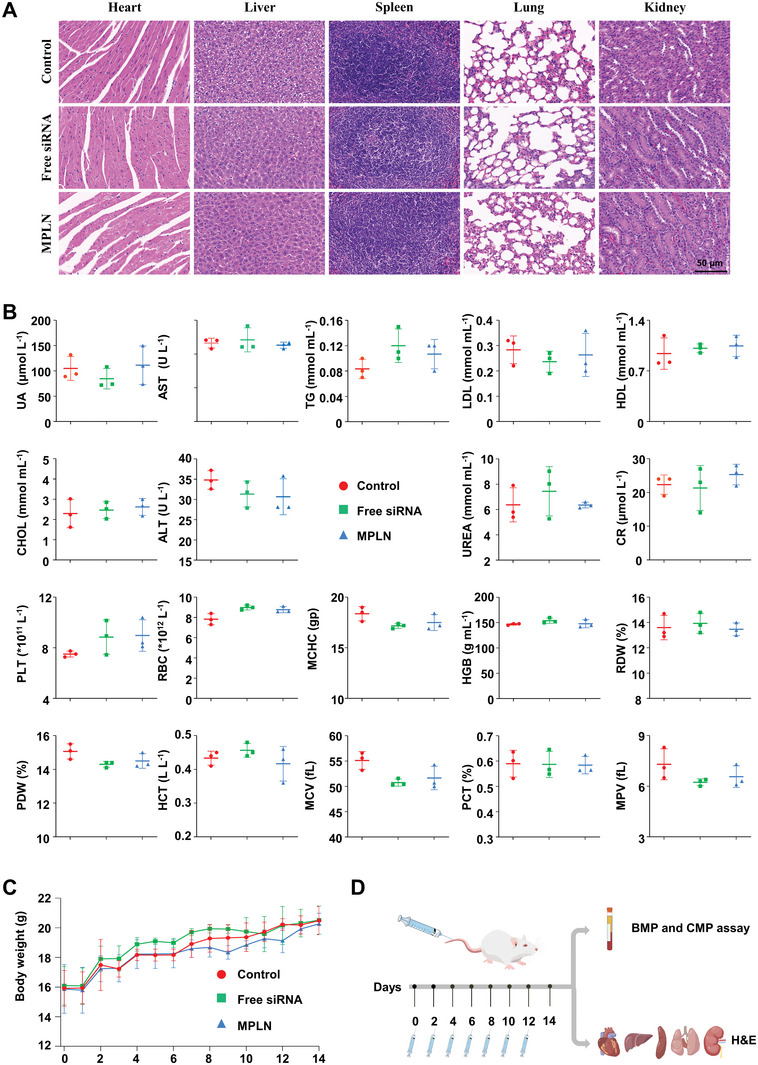
Safety evaluation of MPLN in mice. A) Photographs of the main organs stained with hematoxylin and eosin (H&E). The main organs of the PBMC humanized mice were extracted on the 14^th^ day after these mice were given physiological saline, free siRNA, or MPLN by intravenous injection every 2 days at a dose of 1.2 mg kg^−1^ converting to siRNA. The scale bar is 50 µm for all images. B) Results of BMP and CMP for the PBMC humanized mice on the 14^th^ day after these mice were given physiological saline, free siRNA, or MPLN by intravenous injection every 2 days at a dose of 1.2 mg kg^−1^ converting to siRNA. Data were presented as mean ± SD, *n* = 3, *p*‐values are calculated using the two‐sample *t*‐test. UA: uric acid; AST: aspartate aminotransferase; TG: Triglyceride; LDL: low‐density lipoprotein; HDL: high‐density lipoprotein; CHOL: cholesterol; ALT: alanine aminotransferase; UREA: urea; CR: creatinine; PLT: platelet count; RBC: red blood cell; MCHC: mean corpuscular hemoglobin concentration; HGB: hemoglobin; RDW: red cell volume distribution width; PDW: platelet volume distribution width; HCT: red blood cell‐specific volume; MCV: mean red cell volume; PCT: platelet crit; MPV: mean platelet volume. C) body weight changes of the PBMC humanized mice after treatment with physiological saline, free siRNA, or MPLN. Data were presented as mean ± SD, *n* = 3, *p*‐values are calculated using the two‐sample t‐test. D) Flow diagram of safety evaluation in the PBMC humanized mice.

Histological sections of the main organs, basic metabolic panel (BMP), comprehensive metabolic panel (CMP), and body weight were examined, and the results are displayed in **Figure** **7**A–C. No pathological changes were observed in any of the organs, and there were no significant differences in BMP and CMP.

### 2.10 In Vivo Distribution

**Figure 8:**
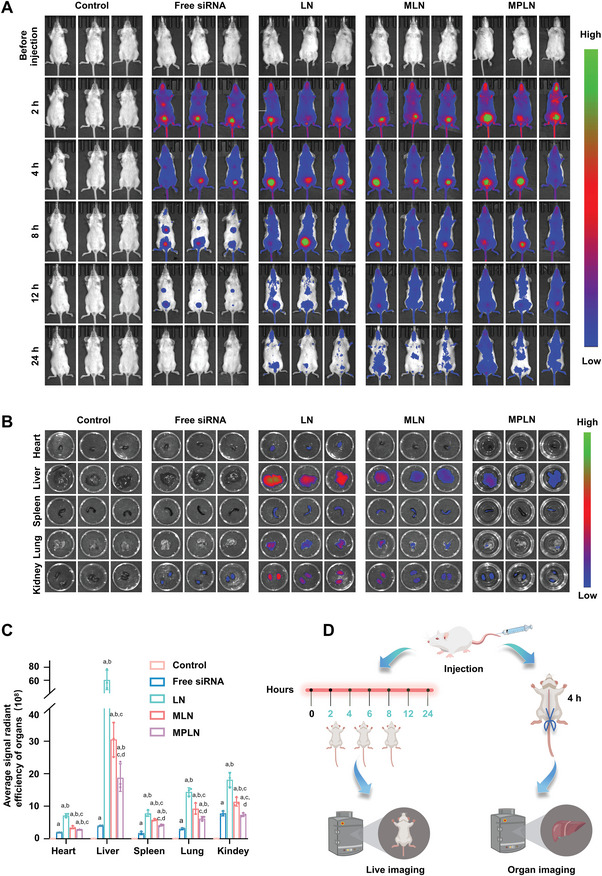
Distribution of Cyanine 7 labeled siRNA in the PBMC humanized mice. A) Systemic fluorescence imaging of the PBMC humanized mice at 0, 2, 4, 8, 12, and 24 h after the single administration of physiological saline, free siRNA, LN, MLN, or MPLN. The experiments were repeated in triplicate. B) Isolated main tissue and organ imaging of the PBMC humanized mice at 4 h after a single intravenous injection of physiological saline, free siRNA, LN, MLN, or MPLN. C) Average fluorescence intensity of the isolated main organs at 4 h after a single intravenous injection of physiological saline, free siRNA, LN, MLN, or MPLN. Data were presented as mean ± SD, *n* = 3, *p*‐values are calculated using the two‐sample *t*‐test. a: *p* < 0.05 versus Control; b: *p* < 0.05 versus free siRNA; c: p < 0.05 versus LN; d: *p* < 0.05 versus MLN. D) Flow diagram of the distribution evaluation in mice.

The distribution of siRNA in mice that received various formulations is displayed in **Figure** **8**A. The mice that received physiological saline showed no fluorescence signal. In mice that received free siRNA, the fluorescence dispersed systematically soon after administration, accumulated gradually in the bladder and kidneys, and finally disappeared completely at 24 h. In mice that received LN, the fluorescence also dispersed systematically soon after administration and accumulated gradually in the bladder and kidneys. Regarding the difference in the distribution of siRNA between the mice that received free siRNA and those that received LN, there was still a weak fluorescence signal observed in the bladder even at 24 h in the mice that received LN. As for the mice that received MLN or MPLN, there was an evident fluorescence signal throughout the body during the entire detection period, and the fluorescence signal in the mice that received MPLN seemed stronger than that in the mice that received MLN.

The distribution of siRNA in the main organs is displayed in Figure 8B. A weak fluorescence signal still existed in the liver, spleen, and kidneys in the mice that received free siRNA, and an obvious fluorescence signal was observed in all of the main organs of the mice that received LN, MLN, or MPLN. As shown in Figure 8C, the fluorescence intensity in the major organs of the mice that received LN was stronger than that in the major organs of the mice that received MLN or MPLN, while the fluorescence intensity in the major organs of the mice that received MLN was higher than that in the major organs of the mice that received MPLN.

## 3 Discussion

MPLN was acquired by introducing 1,2‐distearoyl‐sn‐glycero‐3‐phosphorylethanolamine – N‐methoxy(polyethylene glycol)‐2000 (DSPE‐PEG2000)‐12p1 and the MT2 cell membrane into LN. The insertion of DSPE‐PEG2000‐12p1 had no significant influence on the nanoparticle characteristics, including particle size, zeta potential, and siRNA encapsulation efficacy, while the addition of the MT2 cell membrane evidently raised the particle size and obviously decreased the zeta potential of the nanoparticles. These results are consistent with other literature.^[^ ^6c^ ^]^ The membrane proteins of MPLN were coincident with those of the MT2 cell membrane given that no significant differences were observed in the protein profile, specific proteins, and the protein secondary structure. The results from the colloidal stability experiments showed that both MPLN and its dilutions were stable during the 72‐h period, confirming the in vivo stability of MPLN.

MPLN was considered safe because no obvious cytotoxicity was found in vitro, and there were no evident changes in pathology, BMP, CMP, and body weight in mice treated with MPLN.

The immune‐escape ability of MPLN was identified both in vitro and in vivo. In cellular experiments, the uptake of MPLN by human macrophages differentiated from THP‐1 cells was evaluated both qualitatively and quantitatively. It was obvious that human macrophages more easily ingested LN than those disguised by the MT2 cell membrane. Therefore, the MT2 cell membrane plays an important role in cellular uptake, and the decoration of nanoparticles with the MT2 cell membrane might decrease the clearance by MPS. In the distribution experiments in humanized mice, the encapsulation of siRNA into nanoparticles prolonged the half‐life of siRNA by protecting siRNA from rapid degradation. However, LN, the naked nanoparticles, were almost completely cleared from the humanized mice at 8 h, which was likely due to the nonspecific uptake by MPS because these nanoparticles were mainly distributed in the liver and kidneys at 4 h. In contrast, the nanoparticles disguised with the MT2 cell membrane displayed a longer circulating time, and there was still some siRNA detected in the whole body even at 24 h, especially for MPLN. The amount of siRNA accumulating in the liver and kidneys at 4 h was also significantly decreased for the nanoparticles disguised with the MT2 cell membrane. It is thought that this will be beneficial for the inhibition of HIV because the target cells of HIV are mainly CD4^+^ cells, which are mainly distributed in blood. Taken together, the constructed MPLN possesses obvious immune‐escape ability, which could be attributed to the inherited proteins from the MT2 cell membrane, such as CD47, which is well known as a “don't‐eat‐me” molecule on the autologous cells’ surface and can prevent macrophage‐mediated phagocytosis in MPS. The introduction of PEG2000 was also validated to be beneficial for the immune escape of nanoparticles because PEGylation could form a physical barrier at the surface of the nanoparticles and prevent their subsequent clearance.^[^ ^16^ ^]^

The binding ability and specificity of MPLN with gp120 were validated using HIV‐1 gp120 recombinant proteins, free HIV‐1 virions, and HIV‐1‐infected cells. To exactly reflect the binding ability and specificity of MPLN, two types of HIV‐1 gp120 recombinant proteins and two strains of HIV‐1, representing the X4 tropic strain and R5 tropic strain, were employed. While only slight fluorescence was detected for both LN and EMLN, the introduction of the MT2 cell membrane or 12p1 significantly improved the fluorescence intensity. It was considered as a matter of course that the strongest fluorescence was found in the samples treated with MPLN, which were simultaneously decorated with the MT2 cell membrane and 12p1. The binding specificity of MPLN with gp120 expressed by free HIV‐1 virions was also verified given that the particle size of the mixtures obviously increased compared to that of MPLN or free HIV‐1 particles, and the evident zeta potential changes of the mixtures were also detected. There was a visible overlap between MPLN and HIV‐1 particles. Similarly, strong Cy5 fluorescence was detected in HIV‐1‐infected cells treated with MPLN, while almost no Cy5 fluorescence was found in normal cells treated with MPLN. Therefore, MPLN could powerfully and specifically bind to gp120, regardless of its source and existing status.

MPLN showed a neutralizing breadth of 100% against the combined nine strains of the env‐pseudotyped virus panel and different tropic HIV‐1. The neutralization potency was robust against all nine strains of the env‐pseudotyped virus and different tropic HIV‐1 because the geometric mean IC80 of MPLN against the env‐pseudotyped virus was 29.75 µg mL^−1^, and the IC80 of MPLN was 64.20 µg mL^−1^ for X4‐tropic HIV‐1_NL4‐3_ and 16.39 µg mL^−1^ for R5‐tropic HIV‐1_AD8_. It was obvious that both the IC50 and IC80 of MPLN were much lower than those reported,^[^ ^1^, ^12^ ^]^ and were comparable to those of soluble CD4 and dimeric CD4‐immunoglobulin fusion proteins, which could only partly neutralize the tested env‐pseudotyped HIV tested.^[^ ^3a^ ^]^

It is well known that HIV‐1 primarily infects CD4^+^ T cells, and the infection is initiated by binding the gp120 subunit of env with CD4, which triggers a conformational change in env that allows it to interact with a host cell coreceptor protein CCR5 or CXCR4.^[^ ^3a^ ^]^ Therefore, CD4^+^ T cell‐mimicking nanoparticles were designed for HIV inhibition, and their significant HIV‐1 neutralization was confirmed.^[^ ^1^, ^12^ ^]^ However, these nanoparticles acquired their cell‐mimicking ability by disguising themselves with the membrane extracted from CD4^+^ T cells. Therefore, their evident HIV‐1 neutralization in vivo was unexpected given the high number of CD4^+^ T cells in vivo, which makes it difficult for CD4^+^ T cell‐mimicking nanoparticles to competitively bind with HIV‐1 or HIV‐1‐infected cells.

In this research, 12p1, a peptide that can preferentially bind with gp120 prior to CD4 or CCR5,^[^ ^14^ ^]^ was simultaneously introduced into the novel delivery system based on the MT2 cell membrane so as to improve the competitive strength compared to naive CD4^+^ cells in vivo and thereby enhance HIV‐1‐neutralization ability. The env‐pseudotyped HIV neutralization and HIV‐1 neutralization results confirmed that the nanoparticles decorated with the MT2 cell membrane or 12p1 had significant neutralization abilities for both env‐pseudotyped HIV and HIV‐1. Additionally, MPLN decorated with both the MT2 cell membrane and 12p1 showed the strongest HIV‐1 neutralization, indicating that the combination of the MT2 cell membrane and 12p1 significantly improved the HIV‐1‐neutralization ability. A higher gp120‐binding ability of MPLN than that of MLN or EMPLN was also observed. The enhanced HIV‐neutralizing ability of MPLN may be attributed to the strengthened gp120 binding ability caused by the combination of the MT2 cell membrane and 12p1.

It is well known that siRNA plays its therapeutic role only in the cytoplasm.^[^ ^6c^ ^]^ However, most foreign substances including carriers encapsulating siRNA, are internalized into endocytic vesicles and finally degraded in lysosomes. In other words, endosomal escape is a significant bottleneck in the delivery of therapeutics within a cell, and failure to escape from a lysosome may lead to degradation in the lysosome, especially for siRNA.^[^ ^17^ ^]^ Therefore, the endosomal escape of siRNA was also investigated in this research. Evident endosomal escape of siRNA was confirmed by the abundance of solitary green fluorescence and low levels of yellow fluorescence, presenting the confluence of cyanine 5 labeled siRNA (Cy5‐siRNA) and endolysosomes. The endosomal escape of siRNA could mainly be attributed to the introduction of protamine,^[^ ^18^ ^]^ a widely used and well‐characterized cationic protein approved by the Food and Drug Administration, which has been extensively applied in condensing negatively charged nucleic acids and improving their transfection efficiency.^[^ ^18^, ^19^ ^]^ These capabilities of protamine are supposedly related to its compact region of positively charged arginine residues.^[^ ^20^ ^]^ However, the addition of protamine might influence the release of siRNA from nanoparticles. It was displayed that there was almost no siRNA released from LN. It was different from above the 10% of siRNA released from the reported liposomes.^[^ ^6^ ^]^ This was supposedly due to the formation of siRNA and protamine complex, who was stable in LN and the large size made these complexes difficult to penetrate the bimolecular film of LN. However, the release of siRNA from MLN and MPLN was significantly improved, which might be caused by the introduction of the MT2 cell membrane because the cell membrane possessed negative charges, could competitively bind with protamine, and thereof liberated siRNA from the complexes. The complete release of siRNA from MPLN was observed, and this was believed related to the further addition of DSPE‐PEG2000‐12p1, which had a large steric hindrance and might enhance the distance of molecules. All in all, the sustained and complete release of siRNA from MPLN was beneficial for the homing of MPLN and taking effects of siRNA in the target cells.

*Tat* and *rev* genes were silenced to different extents in HIV‐1‐infected cells treated with different formulations containing siRNA, except in the cells treated with free siRNA.^[^ ^7^ ^]^ It is reasonable to conclude that there was no gene‐silencing efficacy of free siRNA because it was almost impossible for it to enter into the cytoplasm to play a direct action.^[^ ^6c^ ^]^ MPLN decorated with the MT2 cell membrane and 12p1 showed the strongest *tat* and *rev* gene silencing, followed by MLN and LN. Evident *tat* and *rev* gene silencing were also observed in cells treated with antibody‐blocked formulations, although the strength of gene silencing was weakened, which was consistent for cells infected with X4‐tropic HIV‐1_NL4‐3_ or R5‐tropic HIV‐1_AD8_. The most distinguished gene silencing of MPLN was supposedly related to the decoration of the MT2 cell membrane and 12p1, which could promote the binding of the nanoparticles with HIV‐1‐infected cells and increase the amount of siRNA entering the target cells.

Although obvious gene silencing of *tat* and *rev* genes was exhibited after HIV‐1‐infected cells were treated with siRNA encapsulating MLN or MPLN, there was no significant difference in the neutralization or inhibition of HIV‐1 for MPLN encapsulating *tat*/*rev* siRNA or scrambled siRNA. This may be due to the complex action mechanism of siRNA and the long lag time of effects. Indeed, the lag periods between the peak plasma concentrations and peak gene silencing or therapeutic efficacy have been found to range from several days to several months in the clinic for siRNA preparations, including patisiran, cemdisiran, and vutrisiran.^[^ ^21^ ^]^This lag time is believed to be mainly attributed to the slow RNA‐induced silencing complex (RISC) loading process in target cells, which is essential for the specific binding of siRNA to the target complementary mRNA. The gradual loading of RISC is further considered to result from slow endosomal trafficking and the release of siRNA into the cytosol. These processes finally impact pharmacodynamic properties as RISC‐loaded siRNA concentrations have been shown to be directly correlated with kinetics and the magnitude of target protein suppression.^[^ ^21b,c^ ^]^ It is reasonable to suppose that MPLN encapsulating *tat*/*rev* siRNA did not have stronger neutralizing or inhibiting efficacy against HIV‐1 compared to MPLN encapsulating scrambled siRNA because nanocarriers possessing gp120 receptors, such as MLN and MPLN, can rapidly bind to gp120 proteins and directly neutralize HIV‐1 utilizing 12p1 and CD4 molecules at the surface of carriers, but has to spend several days to months to take effect for siRNA due to its complex action mechanism.

It is known that envelope glycoproteins including gp120 can bind to their cellular receptors and chemokine coreceptors prior to viral fusion and entry, and cause bystander CD4^+^ T‐cell death.^[^ ^12^, ^22^ ^]^ This is considered a critical element of HIV pathogenesis given that it contributes to the selective depletion of CD4^+^ T cells and leads to immunodeficiency.^[^ ^12^, ^22^ ^]^ The constructed MLN, MPLN, and MPLN with scrambled siRNA markedly improved the viability of bystander T cells. MPLN and MPLN with scrambled siRNA showed stronger inhibition of bystander T‐cell killing than MLN, while there was no significant difference in the inhibition efficacy caused by MPLN or MPLN with scrambled siRNA. These data indicated that the inhibition of HIV‐1 gp120‐induced bystander T‐cell killing was mainly related to the binding ability with gp120, instead of siRNA.

## 4 Conclusion

In this study, we successfully constructed a lymphocyte membrane‐ and 12p1‐dual‐ functionalized siRNA delivery lipid nanoparticle system. The constructed nanoparticles exhibited an appropriate particle size, good colloidal stability, and satisfactory lymphocyte membrane features. More importantly, this multifunctional nanocarrier achieved evident immune escaping capability, potent gp120 binding ability, robust HIV‐1 neutralization, striking HIV‐1 *tat* and *rev* gene silencing, and obvious inhibition of HIV‐1 gp120‐induced bystander T‐cell killing on the premise of safety. Therefore, satisfactory HIV‐1 therapy efficacy, including HIV‐1 neutralization, specific siRNA delivery into HIV‐1‐infected cells, and reduction of CD4^+^ T‐cell depletion, is expected, and this new system holds potential for HIV‐1 inhibition.

## 5 Experimental Section

### Materials

Hydrogenated soybean phosphatidylcholine (HSPC), and DSPE‐PEG2000 were bought from Shanghai Advanced Vehicle Technology Pharmaceutical LTD (Shanghai, China). DSPE‐PEG2000‐12p1 was prepared by Xi'an ruixi Biological Technology Co., Ltd (Xi'an, China). Lyophilized *tat*/*rev* siRNA (sense strand: 5’‐GCG GAG ACA GCG ACG AAG AGC dTdT‐3’; antisense strand: 5’‐dTdT CGC CUC UGU CGC UGC UUC UCG‐3’), scrambled siRNA (sense strand: 5’‐GGG AGC ACA GGC GCA GAC AGA dTdT‐3’; antisense strand: 5’‐dTdT CCC UCG UGU CCG CGU CUG UCU‐3’), Cy5‐siRNA and Cyanine 7 labeled siRNA were all bought from GenePharma (Shanghai, China). RPMI 1640 and FBS were acquired from GIBCO, Invitrogen (Carlsbad, USA). Antibodies of CD4, CCR5, CXCR4, CD47, and horseradish peroxide (HRP)‐labeled goat/anti‐rabbit IgG were bought from Abcam (Cambridge, UK). R5‐tropic HIV‐1_BaL_ and X4‐tropic HIV‐1_MN_ gp120 recombinant proteins were bought from Sino Biological Inc. (Beijing, China). DiI and Hoechst 33258 were obtained from Beyotime Biotech Inc (Shanghai, China). THP‐1 cells and RAW264.7 cells were obtained from the Cell Resource Centre (IBMS, CAMS/PUMC, Beijing, China). PBMC and human naive CD4^+^ T cells were gifted from OriCells (Shanghai, China). MT2 cells, TZM‐bl cells, and erythrocytes were acquired from State Key Laboratory of Pathogen and Biosecurity (Beijing, China). Env‐pseudoviruses (AE34, B14, B16, B121, BC28, BC29, BC43, C11, and C15), HIV‐1_NL4‐3_, and HIV‐1_AD8_ were all acquired from State Key Laboratory of Pathogen and Biosecurity, Beijing Institute of Microbiology and Epidemiology (Beijing, China). Male PBMC humanized mice (4–5 weeks) were purchased from Shanghai Model Organisms Center, Inc. (Shanghai, China). All animal experiments were performed according to the code of ethics defined by the Animal Care and Use Ethics Committee of the Beijing Institute of Pharmacology and Toxicology.

### Extraction of Cell Membrane

The cell membrane of MT2 cells, a type of lymphocyte, was acquired in accordance with a previously reported method.^[^ ^1^, ^6^, ^12^ ^]^ In brief, the collected MT2 cells were first resuspended in cool PBS (pH7.4) containing ethylenediaminetetraacetic acid (1 µM) and phenylmethylsulphonyl fluoride (1%, W/V), before breaking into pieces in an ice bath by ultrasound with the power of 150 W for 2 min. After incubation at 4 °C for 30 min, the suspension was centrifuged at 500× *g* for 10 min. The obtained supernatant was further centrifuged at 20 000× *g* for 20 min twice, the MT2 cell membrane was acquired after the supernatant was finally centrifuged at 100 000× *g* for 50 min, and the sediments were washed with PBS. The acquired precipitate was finally collected, identified, quantified, and stored at −20 °C for use.

The erythrocyte membrane was isolated following a method similar to that for the lymphocyte membrane.^[^ ^23^ ^]^ Briefly, the collected erythrocytes were treated with precooled PBS containing ethylenediaminetetraacetic acid (1 µM) and phenylmethylsulphonyl fluoride (1%, W/V) for 2 h, and then the suspension was centrifuged at 9050× *g* for 15 min. The acquired precipitate of the erythrocyte membrane was finally collected, identified, quantified, and stored at −20 °C for use.

### Preparation of Various Nanoparticles

LN was constituted with HSPC, cholesterol, and DSPE‐PEG2000 (80:10:10, W/W/W), and prepared via a thin‐film dispersion method with some modifications.^[^ ^6c^ ^]^ Briefly, the lipids were dissolved in chloroform, and the solvent was evaporated under vacuum by a rotator RE‐2000 (Ya Rong Biochemical Instrument Factory, China) at 40 °C for 30 min. The formed thin film was then hydrated with a hydroxyethyl piperazine ethanesulfonic acid buffer solution (20 mM hydroxyethyl piperazine ethanesulfonic acid, 150 mM NaCl, pH 7.0) containing the complex of siRNA and protamine (1:1, W/W) at 55 °C for 40 min, before dispersing the suspension with an ultrasound transducer at 427.5 W for 60 s. Finally, LN was acquired after free siRNA was removed by dialysis. MLN was acquired after mixing LN with the extracted MT2 cell membrane and subsequently extruding through polycarbonate membranes with pore sizes of 400 and 200 nm 20 times respectively. EMLN was prepared using the same method as the MLN. MPLN were finally obtained by inserting DSPE‐PEG2000‐12p1, whose synthetic route and identification are displayed in Figure S1, Supporting Information, into MLN at 37 °C for 30 min,^[^ ^6c^ ^]^ and EMPLN were acquired by inserting DSPE‐PEG2000‐12p1 into EMLN.

### Morphology of Various Nanoparticles

Different nanoparticles were morphologically characterized using TEM (JEM‐1010, JEOL, Tokyo, Japan) and AFM (Bruker Multimode 8, Bruker Daltonic, Billerica, MA, USA).^[^ ^6c^ ^]^ The suspensions of various nanoparticles were dropped on a copper grid, dried at 25 °C, and negatively stained with phosphotungstic acid (2%, W/V) before being observed under TEM. The suspensions of various nanoparticles were spread onto a mica sheet and dried at 25 °C before observing under AFM.

### Particle Size and Zeta Potential of Various Nanoparticles

The particle size and zeta potential of different nanoparticles were analyzed with a Malvern Zetasizer (Nano‐ZS90, British) at 25 °C.^[^ ^6c^ ^]^ The measurements were conducted with 11 cycles, and the results of particle size were shown as Z‐average diameter.

### Encapsulation Efficiency

To calculate the encapsulation efficiency of siRNA in various nanoparticles, the amounts of total siRNA in these suspensions and free siRNA unencapsulated in the nanoparticles were both determined.^[^ ^6^, ^24^ ^]^ To obtain the total amount of siRNA in various formulations, nanoparticle suspensions (200 µL) were first dissolved by adding Triton solution (200 µL, 10%, W/V), and the content of Cy5‐siRNA was measured using a spectrofluorometer with 649 nm as the excitation wavelength and 680 nm as the emission wavelength. The amount of free Cy5‐siRNA in the suspensions was detected with the spectrofluorometer after nanoparticle suspensions (200 µL) were diluted to 2 mL with distilled water, added into the ultra‐filter (vivaspin2; Sartorius biotech, USA), and centrifuged at a speed of 14 000× *g* for 10 min.

The encapsulation efficiency was calculated according to Equation (1), where the *W_total drug_* represents the total amount of siRNA in nanoparticle suspensions and the *W_free drug_* indicates the amount of free siRNA unencapsulated.

(1) $$Encapsulation\;efficiency\left(\%\right)=\frac{W_{total\;drug}-W_{free\;drug}}{W_{total\;drug}}\times 100\%$$

### Protein Detection

To validate the complete transfer of proteins from the MT2 cell membrane onto the nanoparticles, the protein profiles in the purified MT2 cell membrane and MT2 cell membrane‐coated nanoparticles were analyzed using sodium dodecyl sulfate‐polyacrylamide gel electrophoresis.^[^ ^6^, ^25^ ^]^ In brief, the proteins were extracted from the samples with the cell total protein extraction kit, and then separated on a bis‐tris 10‐well minigel (4–12%, W/V) in a running buffer using a Bio‐Rad electrophoresis system at 80 V for 0.5 h and thereafter at 120 V for 1 h. The resulting polyacrylamide gel was finally stained with SimplyBlue overnight for visualization. LN without the cell membrane was analyzed as a negative control.

Specific proteins including CD4, CCR5, CXCR4, and CD47 in the extracted MT2 cell membrane, LN, and MPLN were identified using WB analysis. Briefly, the extracted proteins were first quantified with the Pierce BCA protein assay (ThermoFisher, USA), before separating on an acrylamide gel (10%, W/V) and transferred to a polyvinylidene difluoride membrane (Millipore, USA). Thereafter, the samples were treated with primary antibodies against CD4, CCR5, CXCR4, and CD47 followed by incubating with HRP‐labeled goat/anti‐rabbit IgG. The protein signals were finally detected using a ChemiDoc MP imaging system (Bio‐Rad, USA).

### CD Analysis

To ensure the consistency of proteins from the MT2 cell membrane, MLN, and MPLN in the secondary structure, CD analysis was conducted on a Chirascan‐Plus CD spectrometer with a 0.1‐cm quartz cell.^[^ ^25^, ^26^ ^]^ The concentrations of proteins in different samples were maintained at approximately 0.3 mg mL^−1^. The analysis was performed with a wavelength range of 190–300 nm and a bandwidth of 5 nm. Each spectrum was reported as the average of three scans.

### In Vitro Release

The release of Cy5‐siRNA from LN, MLN, and MPLN was inspected using a dialysis technique at 37 °C.^[^ ^6^ ^]^ Briefly, different nanoparticles (0.5 mL) were added into a dialysis bag (MWCO 50 kDa), immersed in PBS (pH 7.4, 35 mL), and stirred at 100 rpm. Then, the release sample (0.7 mL) was taken out and replaced with an equal volume of fresh release medium at 1, 2, 4, 7, 14, 24, and 48 h. The content of siRNA in the samples was determined using a spectrofluorometer with 649 nm as the excitation wavelength and 680 nm as the emission wavelength. The cumulative release of siRNA was calculated according to Equation (2), where *V* is the volume of release medium, *C_t_* represents the determined concentration of siRNA in the collected samples at time *t*, *∑C_m_* denotes the concentration sum of the collected samples, *V_r_* corresponds to the volume of samples removed for analysis, and *Dose* is the amount of siRNA added into the release medium.

(2) $$Cumulative\;release\left(\%\right)=\frac{\left(V\times C_{t}+V_{r}\times \sum C_{m}\right)}{Dose}\times 100\%$$

### Colloidal Stability

The colloidal stability of MPLN and its dilutions with PBS (pH 7.4) or complete culture medium (RPMI 1640 containing 10% (W/V) FBS) was determined with Turbiscan Lab^®^ Expert at 37 °C, and the evaluating indicators were the changes of transmission and backscattering.^[^ ^6^, ^24^ ^]^

### Cytotoxicity

The cytotoxicity of MPLN at different concentrations was evaluated in TZM‐bl, MT2, and PBMC cell lines.^[^ ^6^, ^25^, ^27^ ^]^ Briefly, TZM‐bl and MT2 cells (6 × 10^3^ cells/well) were first seeded in 96‐well plates, respectively. PBMC (1 × 10^4^ cells/well) were seeded in 96‐well plates and co‐cultured with the stimulating agent phytohemagglutinin (5 µg mL^−1^).^[^ ^28^ ^]^ After incubation for 24 h and rinsing with PBS, the cells were treated with MPLN at siRNA concentrations of 50, 150, 250, 350, 550, and 750 nM for another 24 h. The nanoparticle mass concentrations switched from the siRNA concentration were 0.17 mg mL^−1^, 0.50, 0.84, 1.17, 1.84, and 2.50 mg mL^−1^, respectively. After washing with PBS three times, the cells in each well were further incubated with the cell culture media (100 µL) containing CCK‐8 agent (10 µL) for 2 h. The absorbance of the samples at the wavelength of 450 nm was measured with 650 nm as the reference wavelength. The viability of the cells in the culture medium was defined as 100%.

### In Vitro Cellular Uptake

The in vitro cellular uptake was evaluated in macrophages derived from THP‐1 cells with CLSM (UltraVIEW Vox, PerkinElmer, USA) qualitatively and FCM (BD FACSCalibur, Franklin Lakes, NJ, USA) quantitatively.^[^ ^6^, ^27^ ^]^

For CLSM analysis, macrophages, derived from THP‐1 cells (2 × 10^5^ cells/well) under the action of phorbol ester (200 ng mL^−1^), were seeded on a Petri dish, and cultured for 24 h. Then, the media were replaced with 2 mL of samples containing Cy5‐siRNA (250 nM). After incubation for another 6 h, the cells were fixed with paraformaldehyde (4%, W/V) for 20 min and stained with Hoechst 33258 for 10 min at ambient temperature. Finally, fluorescent images were observed under CLSM.

For FCM analysis, macrophages derived from THP‐1 cells were seeded, cultured, and treated with different samples as in the CLSM analysis. Finally, the cells were detached with Accutase solution and measured with FCM after resuspension in PBS (0.3 mL).

### Binding of MPLN with gp120

To evaluate the binding ability of MPLN with HIV‐1 gp120 molecules, solution (100 µL) containing R5‐tropic HIV‐1_BaL_ or X4‐tropic HIV‐1_MN_ gp120 recombinant proteins (2 µg mL^−1^) was added into each well of 96‐well plates.^[^ ^12^, ^29^ ^]^ After incubating at 4 °C overnight for coating, the solution in each well was removed, and the wells were washed with PBS containing Tween 20 (0.5%, W/V). Then, the wells were incubated with a blocking solution (200 µL) containing normal goat serum (4%, W/V) for 2 h. Thereafter, samples with different concentrations of Cy5‐siRNA were added to the wells. After another incubation for 2 h, the samples in each well were removed, and the plates were rinsed. The fluorescence intensity of the samples at 649 nm with an emission wavelength of 680 nm was finally measured with a Spark microplate reader (Tecan, Männedorf, Switzerland).

To intuitively evaluate the binding ability of MPLN with gp120 on free HIV‐1 virions, the morphology of MPLN, X4‐tropic HIV‐1_NL4‐3_ particles, R5‐tropic HIV‐1_AD8_ particles, and the mixtures of MPLN with free HIV‐1_NL4‐3_ or HIV‐1_AD8_ particles was observed using TEM, and their particle sizes and zeta potential were monitored with a Malvern Zetasizer.^[^ ^12^ ^]^

To validate the binding ability of MPLN with gp120 expressed on HIV‐1‐infected cells, TZM‐bl cells were first infected with HIV‐1_NL4‐3_ (0.01 multiplicity of infection, MOI) and HIV‐1_AD8_ (0.01 MOI). After identification of gp120 expression, the infected cells were incubated with different samples containing Cy5‐siRNA (125 nM) for 2 h. After rinsing, the cells were fixed with paraformaldehyde (40 mg mL^−1^) for 20 min and stained with Hoechst 33258 for 10 min at ambient temperature. Finally, the fluorescence images were visualized under CLSM.^[^ ^29^ ^]^

### HIV‐1 Neutralization

The neutralization breadth and potency of MPLN were first evaluated using pseudovirus and a single round of replication in TZM‐bl cells.^[^ ^12^, ^29^ ^]^ The pseudovirus panel of nine geographically and genetically diverse env‐pseudoviruses (AE34, B14, B16, B121, BC28, BC29, BC43, C11, and C15) representing the main subtypes and recombinant forms circulating in China were used in this paper. Pseudoviruses were obtained via the cotransfection of HEK 293T cells with an env‐expressing plasmid and an env‐deficient genomic backbone plasmid (pSG3ΔEnv) using polyethyleneimine. The 50% tissue culture infectious doses (TCID50) were measured with a luciferase‐based assay in TZM‐bl cells. In brief, serial dilutions of MPLN were incubated with pseudovirus (200 TCID50) in the presence of DEAE‐dextran (15 µg mL^−1^) for 2 h, and the mixtures were further added into TZM‐bl cells (1.0 × 10^4^ cells/well) and incubated for 6 h. After washing with PBS, TZM‐bl cells were incubated for another 48 h, and the neutralizing activity of MPLN was assessed by determining luciferase activity using a FilterMax F5 multimode microplate reader (Molecular Devices). Dose–response curves were fitted using nonlinear regression, and the IC50 and IC80 values were calculated using GraphPad Prism 8.

To further validate the HIV‐1‐neutralization potency of MPLN, two distinct HIV strains, that is, X4‐tropic HIV‐1_NL4‐3_ and R5‐tropic HIV‐1_AD8_, were also used for HIV‐1‐neutralization assay. Briefly, serial dilutions of MPLN were incubated with 200 TCID50 of different viruses respectively in the presence of DEAE‐dextran for 2 h, and the mixtures were further added into TZM‐bl cells (2.5 × 10^4^ cells/well) and incubated for 6 h. After rinsing, the cells were incubated for another 48 h, and the neutralizing activity of MPLN was assessed by measuring HIV‐1 p24 production using the Alliance HIV‐1 p24 Antigen ELISA kit (PerkinElmer).

To verify the enhanced HIV‐1‐neutralization activities of MPLN, the HIV‐1 inhibition of LN, MLN, and scrambled siRNA/MPLN with a concentration equivalent to IC80 of MPLN was further evaluated. The HIV‐1 inhibition of MPLN blocked with the antibody mixtures of CD4, CXCR4, and CCR5 (2:2:1, W/W/W) was also evaluated so as to confirm the possible binding site.

### Inhibition of HIV‐1 gp120‐Induced Bystander T‐Cell Killing

To investigate the effect of MPLN on HIV‐1 gp120‐induced killing of bystander T cells, X4‐tropic HIV‐1_MN_ or R5‐tropic HIV‐1_BaL_ gp120 recombinant proteins were incubated with LN, MLN, MPLN, and scrambled siRNA/MPLN at 25 °C for 2 h.^[^ ^12^ ^]^ Following incubation, the mixtures were added to human naive CD4^+^ T cells with a final nanoparticle concentration of 0.1 mg mL^−1^ and a final gp120 concentration of 1 µg mL^−1^. The treated cells were incubated at 37 °C for 24 h, and then further incubated with cell culture media (100 µL) containing CCK‐8 (10 µL) for 2 h. The absorbance of the samples at 450 nm was measured with 650 nm as the reference wavelength. The viability of the cells in the culture medium was defined as 100%.

### Endosomal Escape

To validate the delivery of siRNA into the cytoplasm, the endosomal escape behavior of MPLN was checked using CLSM.^[^ ^30^ ^]^ Briefly, RAW264.7 cells (2 × 10^5^ cells/well) were seeded on a Petri dish and cultured overnight. Then, the cells were incubated with MPLN at an siRNA concentration of 125 nM for 1, 3, and 6 h. After rinsing with PBS, the cells were further incubated with LysoTracker Red for 30 min. Thereafter, the cells were fixed with paraformaldehyde (4%, W/V) for 20 min and stained with Hoechst 33258 for 10 min at ambient temperature. The cells were finally observed under CLSM.

### In Vitro Gene Silencing

To investigate the gene silencing of HIV‐1 following treatment with MPLN, TZM‐bl cells were first seeded and incubated overnight, and then the cells were attacked with X4‐tropic HIV‐1_NL4‐3_ (0.001 MOI) or R5‐tropic HIV‐1_AD8_ (0.001 MOI) for 6 h. After washing with PBS, the cells were further incubated for 4 days and rinsed with PBS again to remove the free virus. Thereafter, the mixtures of HIV‐1‐infected cells and uninfected cells with the same cell number were incubated for 6 h, followed by treatment with the nanoparticles in different formulations at a siRNA concentration of 200 nM. After 8 h, the culture medium was replaced with fresh culture medium, and the cells were further incubated for 48 h (for mRNA assay) or 72 h (for WB assay). After washing, the cells were digested with trypsin, and the sediments generated by centrifugation were collected for analysis.^[^ ^6^, ^24^ ^]^

For mRNA assay, the total RNA was first extracted from the sediments using a Total RNA Extraction kit (DNase I) (GenePool, GPQ1801). Analysis was conducted on a real‐time fluorescence quantitative PCR detection system (BIOER LineGene 9600Plus), and the relative gene expression was quantified using the 2^−ΔΔCt^ method.^[^ ^7a,b^ ^]^ The primers for PCR amplification were as follows: glyceraldehyde 3‐phosphate dehydrogenase forward: CCT CTG ACT TCA ACA GCG ACA C; glyceraldehyde 3‐phosphate dehydrogenase reverse: TGG TCC AGG GGT CTT ACT CC; *Tat* forward: GGA AGC ATC CAG GAA GTC AG; *Tat* reverse: CTT GGC AAT GAA AGC AAC ACT; *Rev* forward: GAG ACA GAG ACA GAT CCA TTC G; *Rev* reverse: AGT TCC ACA ATC CTC GTT ACA A. The reaction parameters were as follows: 95 °C for 5 s and then 60 °C for 30 s for 45 cycles. The specificity was validated using melt curve analysis and agarose gel electrophoresis. SYBR Green was used in this section.

For WB analysis, the transfected cells were first collected and trypsinized. After centrifugation, the extracted proteins in the supernatant were quantified with the Pierce BCA protein assay and diluted to the same concentration. Thereafter, the proteins were separated on an acrylamide gel (10%, W/V) and transferred to a polyvinylidene difluoride membrane. Then, the samples were incubated with primary antibodies against *tat* and *rev* proteins, before incubating with HRP‐labeled goat/anti‐rabbit IgG. The protein signals were finally detected with a ChemiDoc MP imaging system (Bio‐Rad, Hercules, CA, USA).^[^ ^7^ ^]^

### In Vivo Safety Evaluation

The safety of MPLN was also evaluated in PBMC humanized mice.^[^ ^6c^ ^]^ In brief, the mice were first randomly divided into three groups (six per group), and administered physiological saline (control), free siRNA, or MPLN by intravenous injection every 2 days at a dose of 1.2 mg kg^−1^ converting to siRNA. Fourteen days later, approximately a venous blood sample (1.0 mL) was collected from the anesthetized animals for hemogram assay, before sacrificing the mice. The main tissues, including the heart, liver, brain, lung, and kidneys, were harvested and stained with hematoxylin and eosin for subsequent analysis.

### In Vivo Distribution

To understand the behavior of MPLN in vivo, the distribution of MPLN was evaluated with fluorescence imaging in PBMC humanized mice.^[^ ^6c^ ^]^ Briefly, the mice were randomly divided into five groups (three per group) and administered physiological saline (control) or different formulations containing Cyanine 7 labeled siRNA by tail vein injections at a dose of 1.2 mg kg^−1^ converting to siRNA. Subsequently, the fluorescence imaging was conducted with a NightOWL II in vivo imaging system (IVIS Lumina II, PerkinElmer, Waltham, MA, USA) at a predetermined time.

To intuitively observe the distribution of different formulations in the major organs, the other five groups of PBMC humanized mice were treated as mentioned above. After 4 h, the mice were sacrificed via cervical dislocation, and the major organs, including the heart, liver, spleen, lung, and kidneys, were excised and imaged.^[^ ^6c^ ^]^

### Statistical Analysis

Continuous variables are expressed as mean ± SD. Comparisons between the two groups were performed using the two‐sample *t*‐test. In all cases, significance was defined as *p* < 0.05. Statistical analysis was carried out using EXCEL Software.

## Conflict of Interest

The authors declare no conflict of interest.

## Supporting information

Supporting Information

## Data Availability

The data that support the findings of this study are available from the corresponding author upon reasonable request.
