# Supplementary material for: Lymphocyte Membrane‐ and 12p1‐Dual‐Functionalized Nanoparticles for Free HIV‐1 Trapping and Precise siRNA Delivery into HIV‐1‐Infected Cells
Source: Adv Sci (Weinh). 2023 Feb 8;10(10):2300282. doi: 10.1002/advs.202300282 (PMC10074117; doi:10.1002/advs.202300282)
Supplement: Supplementary file 1 — Supporting Information [file ADVS-10-2300282-s001.pdf]

Supporting Information

Lymphocyte membrane- and 12p1- dual-functionalized nanoparticles for free HIV-1 trapping and precise siRNA delivery into HIV-1 infected cells

Jinbang Zhang[#], Jingwan Han[#], Hui Li[#], Zhengyang Li, Pengfei Zou, Jiaxin Li, Te Zhao, Junwei Che, Yang Yang, Meiyan Yang, Yuli Wang, Wei Gong, Zhiping Li, Lin Li*, and Chunsheng Gao*, and Haihua Xiao*

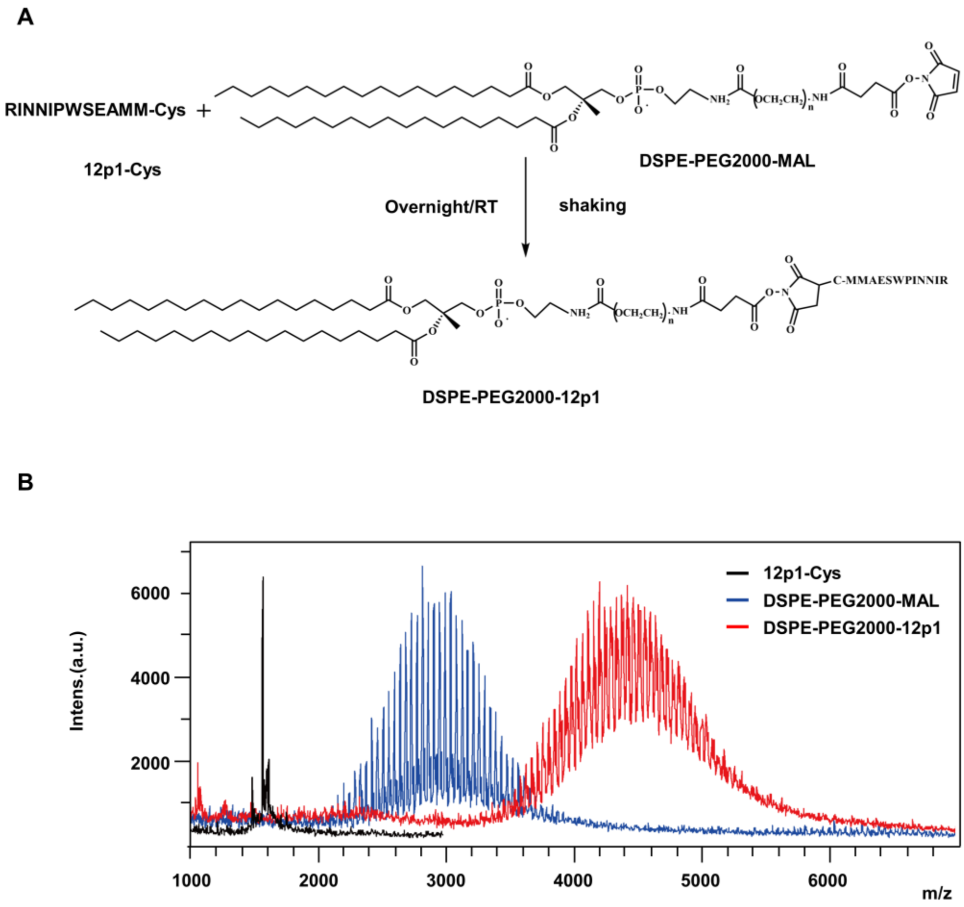

Figure S1. Synthetic route (A) and MALDI-TOF mass spectra (B) of DSPE-PEG2000-12p1.
